# The keybox: Shape-frame fitting during tool use in Goffin’s cockatoos (*Cacatua goffiniana*)

**DOI:** 10.1371/journal.pone.0186859

**Published:** 2017-11-08

**Authors:** Cornelia Habl, Alice Marie Isabel Auersperg

**Affiliations:** 1 Department of Cognitive Biology, University of Vienna, Vienna, Austria; 2 Messerli Research Institute, University of Veterinary Medicine Vienna, University of Vienna, Medical University of Vienna, Vienna, Austria; Fred Hutchinson Cancer Research Center, UNITED STATES

## Abstract

The ability to move an object in alignment to a surface develops early in human ontogeny. However, aligning not just your own body but also the object itself in relation to a surface with a specific shape requires using landmarks rather than the own body as a frame of reference for orientation. The ability to do so is considered important in the development of tool use behaviour in human and non-human animals. Aside from humans, with the exception of a single study on habitually tool using primates, shape-frame matching abilities remain largely unstudied. The Goffin's cockatoo is a generalist parrot, and not a specialised tool user but has shown the capacity to innovate and use different types of tools under controlled settings. We tested these parrots in a tool selection and tool use task featuring objects and their corresponding substrate grooves in a number of shapes with different levels of symmetry. Subjects had to choose the correct ‘key‘ to insert into a box, and align its shape to fit into the corresponding ‘keyhole’ in the box. The parrots were able to select the correct key above chance level from early on in the experiment. Despite their lack of hands, they required fewer placement attempts than primates to insert simple object shapes into corresponding grooves. For complex shapes, they reduced their insertion effort by rotating shapes in their beak while avoiding as many protrusions as possible. Unrewarded play experience with similar object shapes was provided to some of the subjects previously to testing, but did not seem to have an effect on the number of correct choices or on insertion effort.

## Introduction

Fitting an object into a matching outline in a substrate such as inserting a key into a lock or the appropriate screwdriver bit into a screw is a recurrent part of many human technical procedures and, in its simplest forms, develops within the first years of human life. For example, developmental studies on human infants indicate that children have difficulties inserting an elongated object into a slot of similar length before the age of 22 months [1]. Older children start rotating the object into the proper position before bringing it into contact with the slot, indicating that vision plays an important role in their shape-frame matching abilities [1]. In a posting task featuring a round disc and a slot, 18-month-old children were able to orient their own hands but not the disc they here holding in the appropriate way for inserting it [2]. However, they succeeded when the disc was pre-aligned prior to insertion, suggesting that children below the age of 2 have more trouble orienting an object in relation to a substrate than orienting a part of their own body. This is largely because human and non-human animals use two different spatial frames of reference when moving parts of their own body (e.g. a hand) or objects in space: when using an egocentric frame of reference, we move objects relative to our own body, and when using an allocentric one we use environmental objects as landmarks for orientation [3, 4]. Moving an object relative to other objects/substrates, as required when inserting an object into a matching frame is considered to be harder than moving it in relation to one’s own body. Humans start to develop an allocentric frame of reference in the first year of their life, but it improves over a period of several years [5]. Throughout human ontogeny, the improvement of an allocentric frame of reference is also closely linked to the development of tool use [6]. It is likely that being able to use an allocentric frame of reference is an important prerequisite for the development of flexible tool use in animals as well [7].

Another important aspect of shape-frame matching is geometry, as it imposes the number of relations that must be managed simultaneously: a circular object has only one point of reference, whereas a stick-like object with an axis has at least four points that need to be taken into account: two defining the axis of the object and two defining the axis of the groove [7]. Furthermore, a lower degree of symmetry adds to the complexity of the task: a circle has infinite sides of symmetry all going through its centre, a square-shaped object has four sides of symmetry, whereas an equal sided triangle has only three. Hence, in the case of the circular object, as long as you place it in the center of the opening, its orientation does not matter. The square however has 4 possible correct orientations for each front side (turning the object 90° to achieve insertion, and 8 correct orientations turning the object 180° etc.), whereas the front side of a triangle has only 3 (it needs to be turned up to 120°, 6 correct orientations when turned 180°). Correspondingly, according to the Bayley Scales of Infant Development [8], a widely used tool to assess children’s mental, motor and language skills, human infants can insert a ball in a circular opening at 1 year of age, but can only insert a cube into a square opening when they are almost 2 years old. Even more complex are asymmetrical objects, because they can only be inserted in one way and the number of axes that need to be managed relative to the groove is determined by the number of object features added [7, 9]. Fragaszy et al. [9] tested a setup on human infants, in which up to 2 features were added to a single rectangular groove and a matching object: They found that 2, 3 and 4-year-old children consistently aligned a bar-shaped stick and a cross-shaped stick (with one long and one short axis at the top), but had difficulty aligning a tomahawk-shaped stick to a corresponding substrate groove. The two older age classes routinely held the objects above the cutout, comparing it visually before attempting to align it, again suggesting that they did use vision to facilitate their shape-frame matching. When habitually tool using primates, namely capuchin monkeys and chimpanzees, were tested using the same setup [7], both species aligned the long axis of the bar-shaped object with the matching groove more often than expected by chance but with poor precision. Some individuals within both species managed to align the second axis in the cross condition, with subjects making 4 times as many placement-attempts to succeed in the latter alignment than in the original bar condition. Only one capuchin monkey achieved above-chance success at matching 3 features in the tomahawk condition with the corresponding cutouts. Although they do seem to be able to align simple object shapes such as the bar in an efficient manner, habitually tool using primates seemed to perform relatively poorly at this task and apparently lack the visual object guidance of even a 2-year-old human despite having been shown to possess considerable hand-eye coordination [10]. As the above experiment is to our knowledge the only study investigating shape-frame matching in any non-human, we do not yet know whether it evolved as a by-product of digital dexterity in primates or whether it can also develop convergently in other species.

Although most parrots, except for the black Palm cockatoo (*Probosciger aterrimus*) [11,12] are not known to be using tools habitually on a population wide level in the wild, many parrot species playfully insert objects into substrate openings [13–15]. Conspicuously, the pet industry offers various enrichment toys specifically for parrots that resemble simple fitting tasks for human children. During an experiment on object play [14] featuring various differently shaped objects, we were able to repeatedly observe Goffin's cockatoos (*Cacatua goffiniana*) carrying and inserting objects into substrate features inside the aviary that composed nearly an exact frame around the object (S1 Fig). They did so lacking any previous re-enforcement or training. Furthermore, studies on Goffin's cockatoos indicate the capacity for complex forms of tool use and manufacture through innovation in captivity [16] and arguably in a few feral individuals according to unpublished data by Osuna-Mascaro & Auersperg. The tool use of captive individuals is flexibly adjusted depending on the problem at hand [17–19]. Thus, this allows us to investigate shape-frame matching in both unrewarded as well as tool-using contexts for the first time in a non-primate model. Using a series of experiments, we aim to look into several aspects of the shape-frame matching abilities of the Goffin's cockatoo.

In a first instance, we are interested in the behaviour within the context of unrewarded object play. We would like to determine the rate of reoccurrence of playful shape-frame matching and whether this particular type of object play is restricted to specific object shapes and/or to certain individuals. Due to our previous observations a few years ago, we predict that cockatoos will playfully match shapes to frames irregularly and that they will match highly symmetrical shapes such as circles more often than less symmetrical shapes in this context.

Furthermore, we aim to test shape-frame matching in a tool using context using a box featuring several different types of ‘keyholes’ that allow for the insertion of only one out of a selection of 3 or 5 object shapes (‘keys’). The keyholes have different levels of symmetry and differently shaped features.

Within this tool use task, we first want to look at tool selection: Are the birds able to select the correct tool for the keyhole at hand and is performance spontaneous or is there a learning process involved? Whereas the cockatoos have been shown to react flexibly and sensibly in a tool selection task featuring two very different tasks [19], learning a simple virtual matching to sample task on the touch screen was a long and tedious process which did not lead to success in many cases [20]. If the birds are able to select the objects that corresponded to the correct frames, it would indicate that adding physical context to a matching to sample task increases performance, as has been shown in a few other species, e.g. Kea (*Nestor notabilis*) [21], Pigeons (*Columba livia*) [22] and humans [23]. In the second aspect of the keybox task we are interested in the effect of object/frame geometry on insertion success/effort. We would like to establish if the animals only succeed when inserting specific shapes, and within the successful shapes which geometric forms cause most difficulty. Based on previous results on human infants and primates [7, 9] we expect the animals to follow insertion patterns as predicted for the unrewarded object play experience. Finally, we would like to assess the effect of shape-frame matching experience during object play on their performance in the keybox task. One of the benefits of object play is in many cases speculated to be practicing the neuro-muscular system for enhanced performance when confronted with future foraging problems [24, 25], however this has rarely been tested systematically. Demery & Chappell [26] tested Senegal parrots’ (*Poicephalus senegalus*) and kakarikis’ (*Cyanoramphus novaezelandiae*) sensitivity to a change of visible (colour, shape) and invisible (weight) cues during object exploration. Both species explored functional and invisible changes more than non-functional or visible ones, and interestingly, asymmetrical objects more than symmetrical ones. Because object play presents animals with an opportunity to explore objects’ physical properties, we therefore predict an effect of previous object play experience on both the performance in selecting the appropriate tool as well as on insertion efficiency.

## Materials and methods

### Subjects

13 adult, captive- born and hand-reared Goffin’s cockatoos (*Cacatua goffiniana*), participated in this study.

The subjects (8 males, 5 females) were kept in a single-species group at the Goffin Lab of the University of Veterinary Medicine (Vienna, Austria) and were housed in a large, enriched aviary with an indoors and outdoors area (in total ca. 200 m^2^ ground, space up to 6m high). All parrots were kept on an ad libitum diet (fresh and dried fruits, boiled vegetables, a mixture of boiled and raw seeds and fresh water). For further information about the subjects see S1 Table.

#### Experimental history

All subjects had previously been involved in various physical problem-solving tasks [27, 28], including studies involving the use of tools [16–19, 29]. Additionally, five years before the data collection started, in the summer of 2011, all (except for birds hatched in 2011) participated in a monitoring study on object play in which some shape matching actions were accidentally observed in an unrewarded context [13] (S1 Fig). However, they had not experienced the objects and their respective negatives (shaped holes corresponding to the objects) used in this experiment.

#### Ethical note

All animals are permanently kept (before and after the experiment) in a well-established group at the “Goffin Lab”. All have CITES certificates and were registered at the district’s administrative animal welfare bureau (Bezirkshauptmannschaft St. Pölten Schmiedgasse 4–6, A-3100, St. Pölten, Austria). Our housing conditions comply with the Austrian Federal Act on the Protection of Animals (Animal Protection Act—§24 Abs. 1 Z 1 and 2; §25 Abs. 3—TSchG, BGBl. I Nr. 118/2004 Art. 2). As our experiments are strictly non-invasive and based purely on behavioural observations, they are not classified as animal experiments in accordance with Austrian Law (Austria: §2. Federal Law Gazette No. 501/1989) and do not require permission. The birds are not wing-clipped and only enter the experimental room on a voluntary basis. Our animals are never food deprived and are closely monitored on a daily basis; no elevated levels of stress or aggression could be detected throughout our testing period.

### Unrewarded shape matching

In the Unrewarded Shape Matching (USM) section of this study we tested the propensity of our subjects to playfully establish unrewarded shape-matching relationships between objects. This monitoring block additionally served as pre-experience for 7 of the 13 birds in order to assess a possible effect of enhanced play experience on later task performance, which was conducted immediately after the monitoring block had ended.

**Fig 1:**
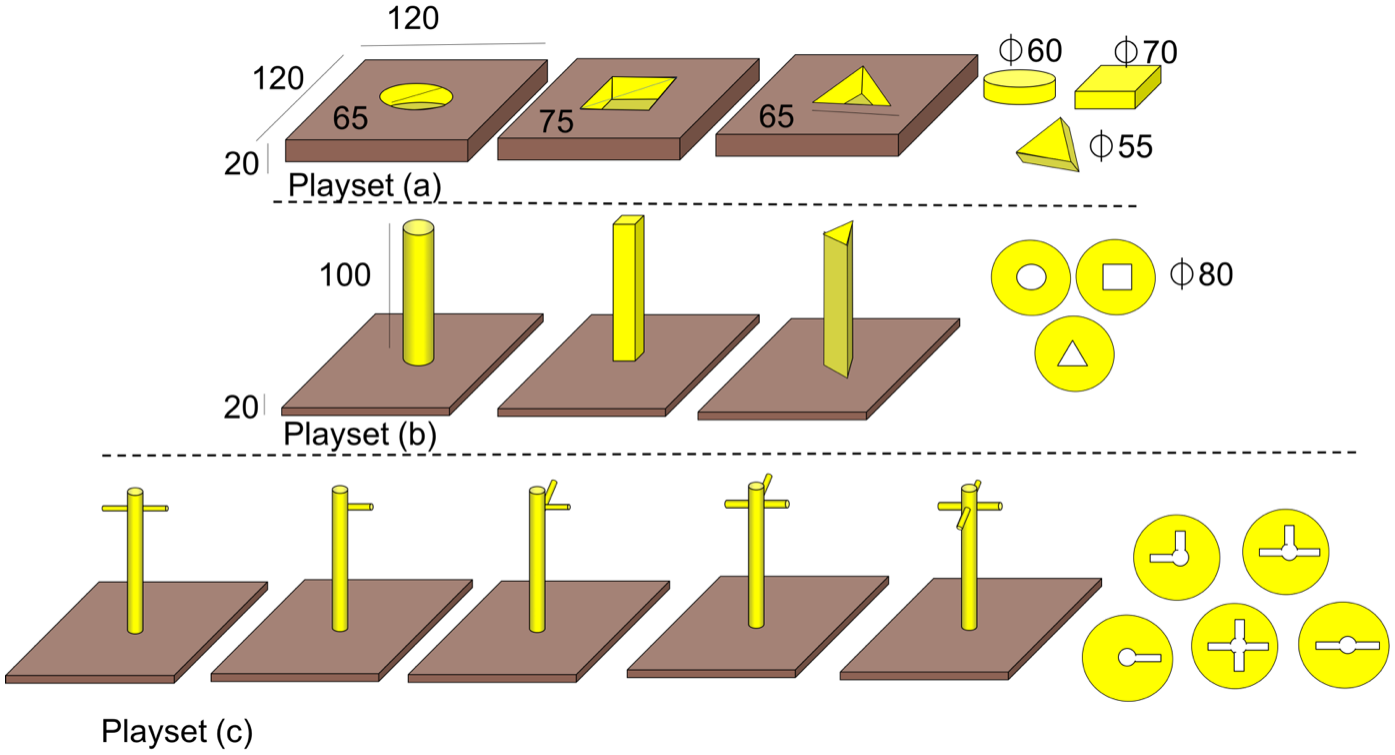
The 3 playsets (a-c) with dimensions. Two of each object shape were offered alongside the poles or indents for each set. Dimensions in mm.

During testing, the 7 subjects were visually isolated from the remaining group and exposed to different playsets (Fig 1).

Each playset consisted of yellow wooden substrate boards with either indentions and their correspondingly shaped free objects, or poles and discs with the corresponding negatives (see Fig 1 for dimensions). The substrate boards were placed next to each other in random order and the objects were scattered around (or pre-stacked/inserted upon/into; see phase 2) them. The wooden playsets were offered in three consecutive phases: in phase 1, the shaped objects were all scattered around the substrates (shaped poles or holes); in phase 2 one set of objects was pre-stacked/pre-inserted onto/into the poles/holes to enhance unstacking behaviour; during phase 3, the subjects received one stacking/unstacking demonstration by a human demonstrator for each substrate (pole/hole) in random order before receiving the setup as in phase 1. Subjects were free to interact with each playset (in the same order A, B, C) in an unrewarded context (each phase consisting of 5 sessions, which lasted for 30 minutes in phases 1 and 2, and 10 minutes in phase 3 respectively)

Within all phases, we scored the frequencies of object-substrate (poles/holes) combinations. Within the latter, three distinctions were made: ‘incorrect’ (combined shapes do not match); ‘correct’ (combined shapes match but are not fully stacked/ inserted) and successful ‘shape-match’ (correct object is successfully stacked/inserted).

### The keybox

#### Apparatus and material

**Fig 2:**
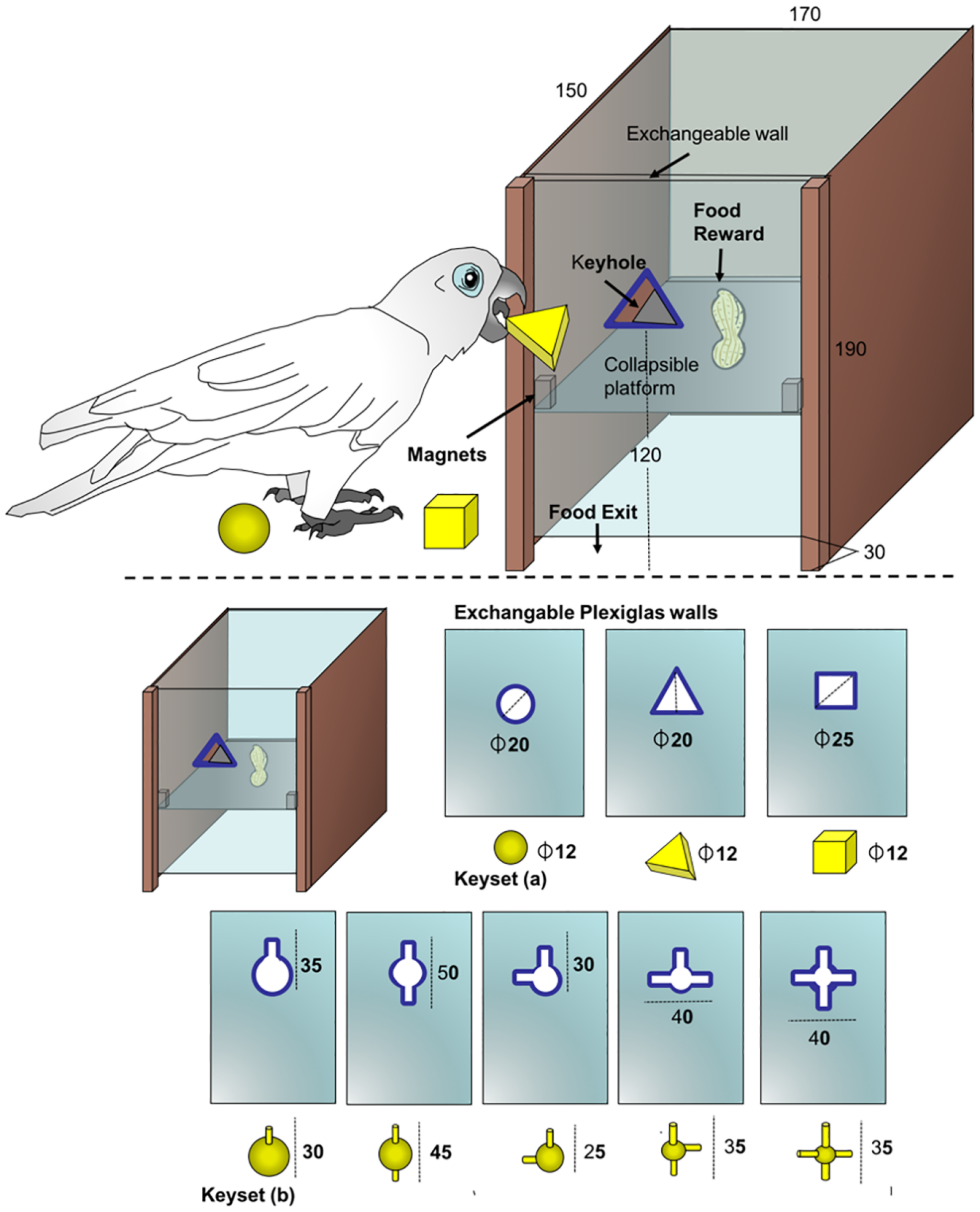
Top: Basic apparatus with dimensions; Bottom: Exchangeable Plexiglas walls with different keyholes and corresponding keys. Each key corresponds only to one keyhole within each keyset (for this reason in keyset B, the diameter of the central key part therefore decreases alongside the complexity of its shape). Dimensions in mm.

The keybox apparatus (see Fig 2 for dimensions) was made from hard beech wood. It had three wooden sides, a Plexiglas^®^ roof and was screwed onto a wooden plate. We used 8 exchangeable Plexiglas^®^ walls in total, because similar shapes as in the USM part were divided into keyset A (3 keys) and keyset B (5 keys; see description in Fig 2). Correspondingly, 3 walls featured ‘keyholes’ corresponding to the shapes of keyset A, and 5 with the shapes of keyset B; the contour of the keyholes were highlighted with black permanent marker. These walls could be slid into guide rails in place of the fourth side and were secured by wood chips between the wall and the roof. Inside the box, a collapsible platform was affixed halfway up the back wall and was held horizontally by a magnet, whose strength was set in a way that any inserted key would cause the platform to collapse. The keys were molded using yellow Fimo^®^ clay (with each the same amount of small lead balls inside to increase the weight, approximately 10 grams) and later baked at 130°C to harden. In order for a specific key to only fit through its respective wall, the keys within keyset B were thickened at their bases (the centre of the shape) and the bases had different diameters (Fig 2; S2 Fig).

#### Procedure

The keybox test series tested for correct tool selection and shape-matching abilities as a means to retrieve food from a box. It consisted of a short training phase followed by a testing phase. For all tests, the individual subjects were visually isolated from their conspecifics when placed into an experimental compartment. During the training phase, birds were given the opportunity to insert a small, randomly shaped natural stone into a Plexiglas^®^ wall of the keybox (round tool opening) to retrieve a food reward. Birds were tested until they retrieved the food on each trial for two consecutive sessions of 10 trials each, with trials lasting a maximum of 10 minutes before testing was discontinued and a new session was started on the following day (note that subjects had pre-experience inserting compact objects into tubes from Laumer et al. [19] and thus had little problems in the transfer).

During the first keybox task, all subjects were presented with keyset A (6 sessions à 12 trials) and keyset B (12 sessions à 10 trials) together with the apparatus. Within each session (12 trials) of keyset A (3 keys), each of the 3 exchangeable walls would be placed into the keybox 4 times (in random order), and within each session (10 trials) of keyset B (5 keys) each wall would be placed into the keybox twice. Hence, each key was the correct choice a total of 24 times throughout the experiment. For both keysets, half of the sessions were first presented as a ‘Spontaneous Choice Condition’: As soon as the bird combined a key with the Plexiglas^®^ front of the keybox, the remaining keys were immediately removed. If subjects chose the correct tool, they had 5 minutes to retrieve the food before the trial was discontinued and the next trial started. If they chose an incorrect key, they had to wait 3 minutes before the next trial started. If a bird failed to choose a key within 10 minutes, the trial was terminated. Thereafter, the keys were presented in a ‘Learning Condition’: All keys remained on the table for a period of 10 minutes or until the food was retrieved, allowing the subject to try out different key combinations within the same trial.

In very few instances, birds either managed to forcefully squeeze a key through a non-matching hole or moved the keybox itself so that the platform collapsed. These cases were not counted as successful, the reward was removed immediately and the trial was terminated.

The keybox was baited out of sight with a piece of cashew nut and thereafter placed onto an experimental table (70x70cm). The subject was placed on the backrest of a chair in front the apparatus. After a five second delay, during which the bird had the chance to look at the respective Plexiglas^®^ wall in the keybox, all keys of the corresponding keyset were placed on the table in a straight line ca. 30 cm parallel to the front of the keybox in random order from left to right. After a second delay of 10 seconds, during which the bird could look at both the keys and the wall, the bird was allowed to pick a key. The experimenter stood behind the camera, wore mirrored sunglasses and avoided lateral head movement throughout the entire testing phase.

The data for the USM was collected between March 2015 and October 2015 and for the keybox test series between November 2015 and June 2016.

### Analysis

All trials were videotaped (JVC Camcorder Model No.: GZ-HM30BE) and coded *in situ* as well as from the videos. We counted all object combinations with the substrate grooves in the USM. Within all object combinations we highlight combinations of objects with the correct frame and actual shape matches. In the keybox test we scored the first object that was combined with the front of the keybox as correct/non-correct; whether the subject was successful in retrieving the food from the platform in this trial, if any objects and how many were lifted before one was combined with the frame (‘switch’) for each keyset (A & B). Furthermore, in trials in which the correct object was used, we scored the duration from the first contact between the object and the box to the insertion (‘duration’) and the number of times it was brought in contact with the opening (‘combination frequency’). The latter two scorings were only conducted for condition 1 in order to look at spontaneous performance.

#### Unrewarded shape matching

We conducted General Linear Mixed Models (GLMMs) in SPSS (Version 24) for each playset (A, B & C) in USM on ‘mean number of correct combinations’ and ‘mean number of shape matches’ as target variables. Random factor was ‘individual’ and fixed factors were ‘object’ (3 or 5 different objects in each playset), ‘phase’ (objects next to substrates; objects prestacked/preinserted; objects next to substrates after human demo) and ‘session’.

#### Keybox: Selection

We conducted a GLMM on ‘mean number of correct keys’ selected for each keyset (A & B). Random factor was ‘individual’, fixed factors were ‘group’ (play experience or not) ‘key’, ‘condition’ (condition1 and 2), ‘session’ and an interaction ‘group*key’.

Furthermore, as subjects frequently picked up an object and discarded it before combining another object with the box, we looked at the number of times this occurred in each trial. Again, we conducted GLMMs on this ‘switch’ of a specific key for each keyset (A & B). Again, random factor was ‘individual’, fixed factors were ‘group’ (play experience or not) ‘key’, ‘condition’ (condition1 and 2), ‘session’ and an interaction ‘group*key’.

#### Keybox: Insertion effort

As it was not possible to reliably score the exact alignment of the object features with the keyhole (the birds were often blocking the full sight towards the keyhole with their body), we decided to use duration and frequency of combinations in order to obtain a measure on which shapes caused most difficulties during insertions. For trials in which the correct object was selected, we conducted GLMMs on mean duration (in seconds; first contact of object with keybox to insertion) and on mean frequency (number of times the object was brought into contact with the opening before successful insertion). Random factor was ‘individual’, fixed factors were ‘group’ (play experience or not) ‘key’, ‘condition’ (condition1 and 2), ‘session’ and an interaction ‘group*key’

We used Wilcoxon signed rank and Mann-Whitney U tests for posthoc analysis, and Binominal tests to assess individual data. We used the Bonferroni-Holms method to control for multiple comparisons.

We controlled for inter-observer reliability by double-scoring of 10% of the data by a naïve coder. The inter-rater correlation coefficient (ICC) suggests perfect agreement between the two raters for (all *ICCs* >0.9, S2 Table) in all relevant measures.

## Results

### Unrewarded shape matching

**Fig 3:**
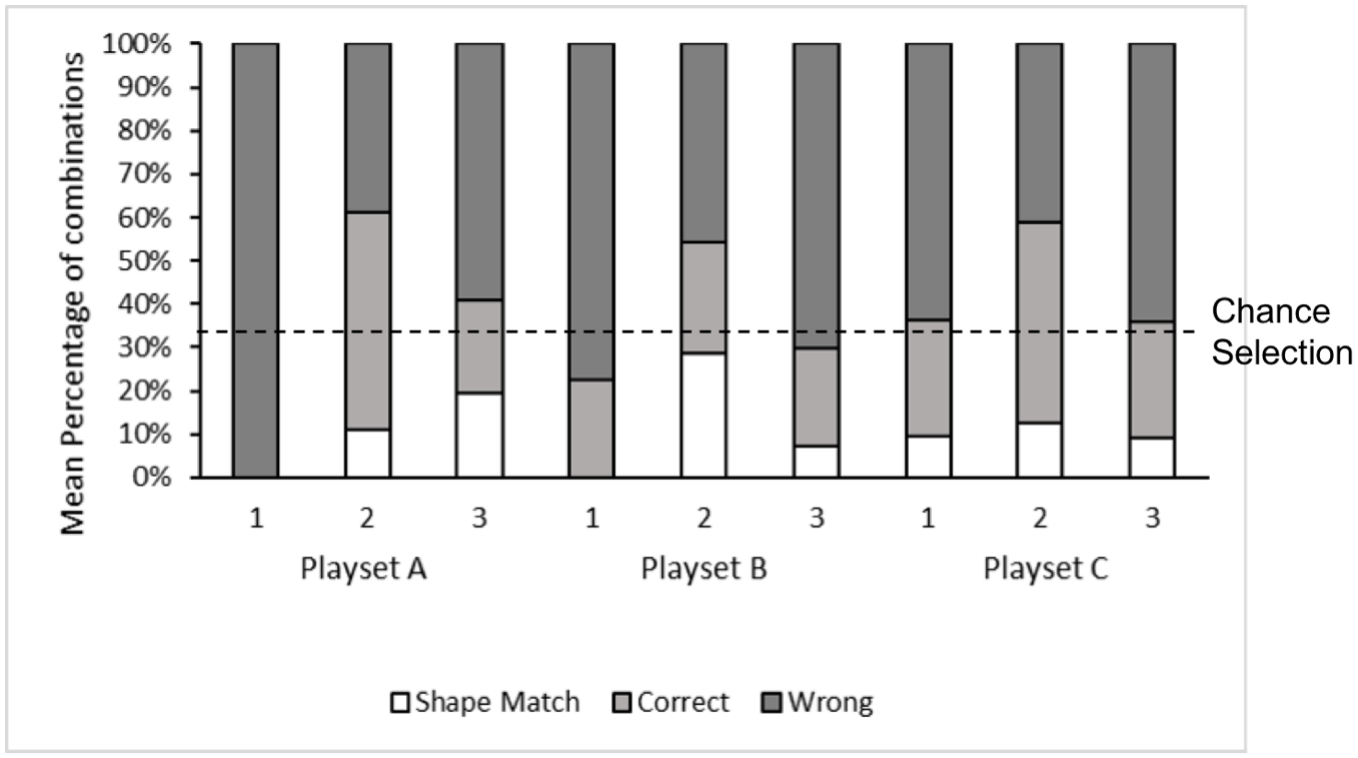
Play: Mean percentage of combinations of objects with substrate grooves. Combinations of an object with the corresponding hole (correct), within corrects, combinations in which the object was fully fitted (shape match) and combinations of objects with different shapes (wrong). Data is shown for each phase (1–3) within each playset (A-C).

For playsets A-C subjects did not combine the object with their corresponding substrate grooves above chance expectation (One-Sample Wilcoxon tests N = 7, p<0.05). Nevertheless, for playsets A and C, the factors ‘phase’ and ‘object’ and, for playset B, the factor ‘phase’ but not any other factors measured had a significant effect on both the number of correct combinations of objects with their corresponding grooves and shape matching (p<0.05; see S3 Table for detailed GLMM results). The factor ‘session’ did not have a significant effect on performance for any of the target variables in any playset (S3 Table). Posthoc tests indicate that birds improved across phases with more correct combinations of objects with corresponding grooves in phases 3 and 2 for playsets A and B (Wilcoxon signed Rank tests N = 7 PSA: Z = -2.207, p = 0.031; PSB: Z = -2.366, p = 0.016; non-sig. trend for PSC: Z = -1.913, p = 0.063; there was no difference between phases 1 and 2 or 2 and 3 for any playset; see Fig 3). The number of shapes matched did not improve above trend levels across conditions (Wilcoxon signed rank tests N = 7 p<0.05) except for playset A, for which we similarly found a difference between phases 1 and 3 (Wilcoxon signed Rank test N = 7, Z = -2.201, p = 0.031). The corresponding posthoc tests did not reveal differences either in the number of birds’ correct object-groove combinations nor shape matches between different types objects for any playset (Wilcoxon signed Rank tests N = 7, p<0.05)

### Keybox

When birds selected the correct object, they were nearly always able to obtain the food reward by inserting the ‘key’ into the ‘keyhole’ within the 5 minutes given (except for 19 trials of 936 in keyset A and 21 trials of 1560 in keyset B).

#### (a) Selection

**Fig 4:**
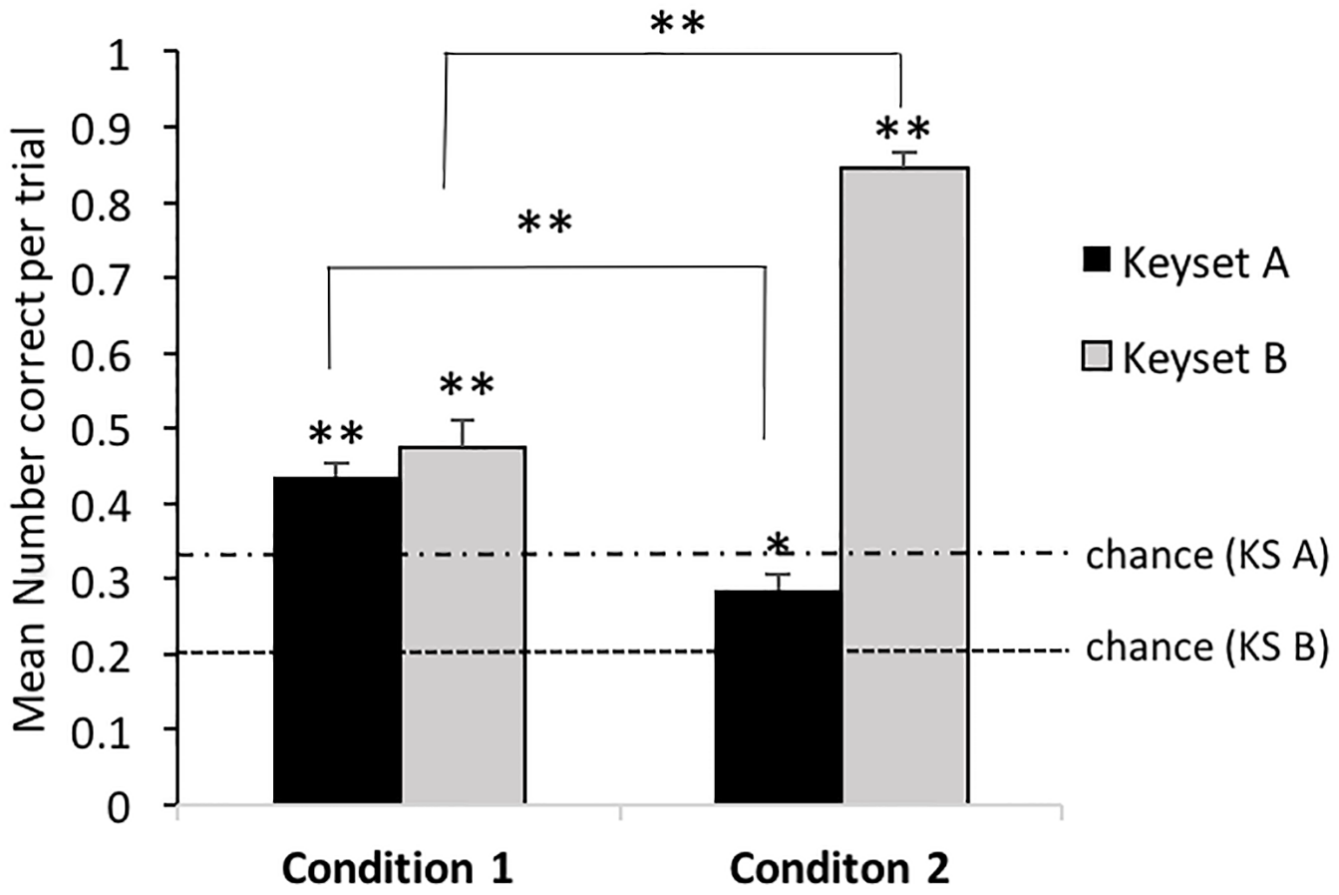
Selection: Mean number of correct choices per trial. Keyset A (black columns) and B (grey columns). Chance expectation for keyset A = 0.33; for keyset B = 0.2 correct per trial. * chosen above chance expectation; ** p<0.0001.

**Fig 5:**
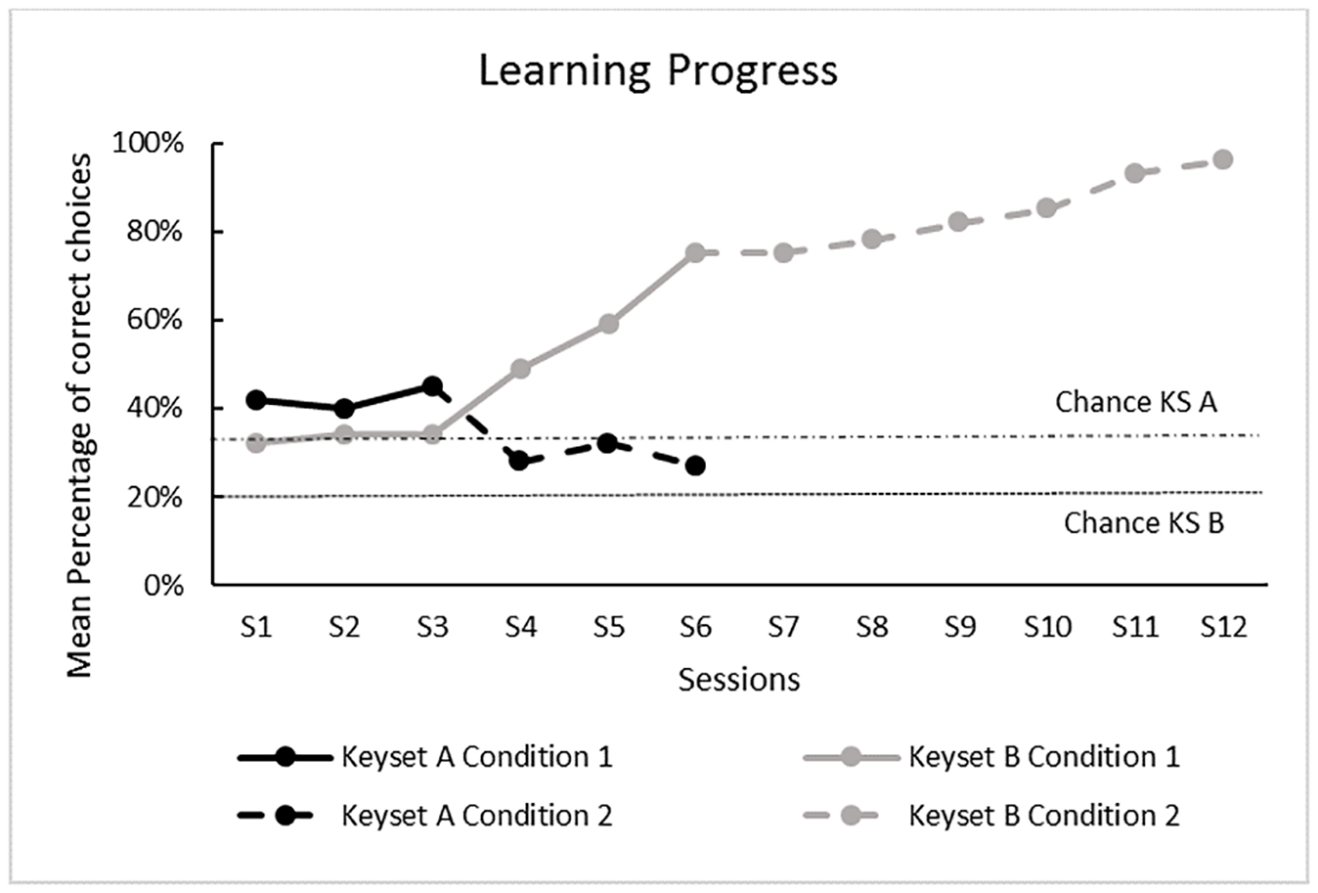
Selection: Percentage of correct choices per trial over sessions. Black line = keyset A; grey line = keyset B; intact grey/black line = condition 1; dashed grey/black line = keyset B; Chance expectation for keyset A = 33% correct; for keyset B = 20% correct.

Subjects already chose the correct key above chance in condition 1 of keyset A (one Sample Wilcoxon Test N = 13, Z = -3.177, p<0.0001) but not in condition 2 (one Sample Wilcoxon Test N = 13, Z = -1.834, p = 0.068M; see Figs 4 and 5). They also chose the correct key above chance in the first 3 sessions of condition 1 within keyset B (one Sample Wilcoxon Test N = 13, Z = -3.194, p<0.0001) and maintained their performance within the same condition (one Sample Wilcoxon Test N = 13, Z = -3186, p<0.0001) and in condition 2 (one Sample Wilcoxon Tests N = 13, Session 1–3 Z = -3186, p<0.0001; Session 4–6 Z = 3.241, p<0.0001; see Figs 4 and 5). See S4 Table for detailed individual data on selection. The GLMMs showed significant effects for the factor ‘condition’ in keyset A (F = 24.05, df1 = 1; df2 = 225; p<0.0001) and B (F = 285.873, df1 = 1; df2 = 764, p<0.0001) and for the factor ‘session’ in keyset B (22.505, df1 = 5, df2 = 764, p<0.0001) but not for any of the other factors measured (see S5 Table for detailed GLMM results). As there were only 2 conditions per keyset, no posthoc tests were required. Surprisingly, the birds selected the correct key more often in condition 1 than in condition 2 for keyset A; the opposite was found for keyset B (Fig 1). When dividing the 6 sessions within keyset B into 2 blocks of 3 sessions for each condition, posthoc tests revealed a steady increase in performance across session blocks (note that, as mentioned early in the Results section, performance was already above chance expectation within the first session blocks). There was an above chance difference between session block 1 and 2 in condition 1 (Wilcoxon signed rank Test N = 13, Z = -3.116, p<0.0001) between session block 2 in condition 1 and session block 1 in condition 2 (Wilcoxon signed rank Test N = 13, Z = -2.914, p = 0.002) and session block 1 and 2 in condition 2 (Wilcoxon signed rank Test N = 13, Z = 2.949, p<0.0001; see Fig 5).

#### (b) Switch

The GLMMs on the ‘switch’ trials showed a significant effect of the factor ‘condition’ for keyset A (F = 39.611, df1 = 1, df2 = 225, p<0.0001) and of ‘key’ for keyset B (F = 2.526, df1 = 4, df2 = 764, p = 0.04) but not for any of the other factors measured (See S6 Table for detailed GLMM results). As there were only 2 ‘conditions’, posthoc tests were unnecessary, they showed a subject mean of 0.27 +/-0.13SE in condition 1 and of 1.102 +/- 0.2 SE in condition 2 of keyset A. For the factor ‘object’ in keyset B we only found significantly more switches before subjects inserted the One-Arm than the L-Shape (Wilcoxon signed Rank Test N = 13, Z = -2.71, p = 0.004) or the Tripod (Wilcoxon signed Rank Test N = 13, Z = -2.98, p = 0.001; subjects means for each shape: One-Arm 1.14+/- 0.08 SE; Two-Arm 0.89+/- 0.1 SE; L-Shape 0.91+/- 0.01 SE; Tripod 0.82+/- 0.09 SE; Cross 0.97+/- 0.08 SE).

When looking at the percentage of switches in which the second object the birds selected was the correct one, we found that the group exchanged their dropped choice for the correct match the keyhole significantly above chance expectation in keyset B (One Sample Wilcoxon test N = 13, Z = 3.18, p<0.001) but not in keyset A (One Sample Wilcoxon test N = 13, Z = 0.94, p = 0.36). Individual data suggests that one bird switched correctly above chance for keyset A and all for keyset B (Binominal Tests p<0.05; See S7 Table).

#### (c) Insertion effort

**Fig 6:**
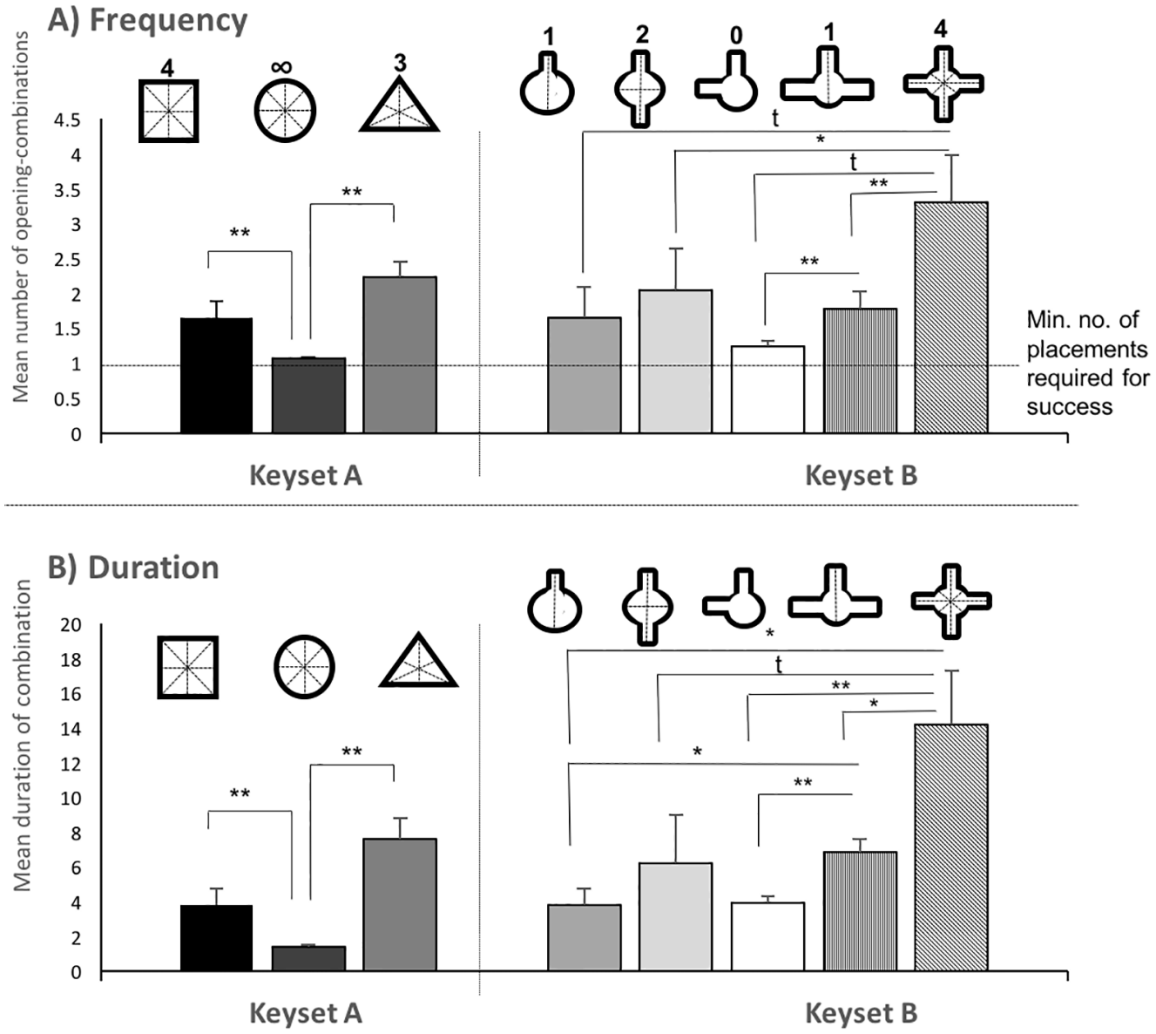
Insertion effort. A) Mean number of combination of a correct key with its corresponding opening before insertion for each key within keyset A and B respectively (numbers above keys represent numbers of sides of symmetry) B) Mean duration (in seconds) of combination of a correct key with its corresponding opening before insertion for keyset A and B respectively. * above chance expectation; ** p<0.0001; t = n.s. trend: p<0.05 but not above chance after Bonferroni-Holms correction for multiple comparisons.

The GLMM on the duration of combining the correct object with the box prior to insertion (in sec) revealed a significant effect of the factor ‘key’ in both keyset A (F = 15.56, df1 = 4, df2 = 355, p<0.0001) and keyset B (F = 16,53, df1 = 2, df2 = 355, p<0.0001) and an effect of an interaction of ‘group*key’ in keyset A (F = 3.24, df1 = 4, df2 = 355, p<0.012) but not for other factors measured (see S8 Table for detailed GLMM results). As predicted we found that the birds took longer to insert the Triangle (Wilcoxon signed Rank Test N = 13, Z = -3.18, p<0.0001) and the Square (Wilcoxon signed Rank Test N = 13, Z = -3.18, p<0.0001) than the Circle within keyset A. The difference between Triangle and Square was not above chance (Wilcoxon signed Rank Test N = 13, Z = -1.083, p = 0.279). For keyset B, we found that subjects took longer to insert the Tripod than the L-Shape and the One-Arm (Wilcoxon signed Rank Test N = 13, L-shape Z = -3.181, p<0.000; One-arm Z = -2.41, p = 0.01) and longer for the Cross than any other object (Wilcoxon signed Rank Test N = 13, L-shape Z = -3.041, p = 0.001; One-Arm Z = -2.55, p = 0.008; Tripod Z = -2.411, p = 0.013, Two-Arm Z = -2.341, p = 0.01; see Fig 6). We did not find a significant effect of ‘key’ on the duration of combination (Mann-Whitney U tests all p<0.025; n.s. after Bonferroni Holms correction).

The GLMM on the number of times the birds combined the correct key with the keyhole before insertion (frequency) similarly indicates a significant effect of key-type for both keyset A (F = 13.071, df1 = 2, df2 = 331, p<0.0001) and keyset B (F = 7.113, df1 = 4, df2 = 335, p<0.0001) but not for any other factor measured (see S8 Table for detailed GLMM results).

Again, we found that the animals combined the Triangle (Wilcoxon signed Rank Test N = 13, Z = -3.06, p<0.0001) and the Square (Wilcoxon signed Rank Test N = 13, Z = -3.018, p<0.0001) more often than the Circle with the corresponding keyhole before succeeding to insert it, but the difference between Triangle and Square was not above chance (Wilcoxon signed Rank Test N = 13, Z = -3.14, p = 0.787; see Fig 6 for more detail). Within keyset B they needed more combinations for the Cross than for the L-shape (Wilcoxon signed Rank Test N = 13, Z = -2.904, p = 0.001) and the Two-Arm (Z = -2.8314, p = 0.002). The frequency difference between the Cross and the Tripod (Z = -2.238, p = 0.023) and the Cross and the One-Arm (Z = -2.132, p = 0.033) was a non-significant trend after a correction for multiple comparisons using the Bonferroni-Holms method. For the Tripod, subjects also required significantly more combinations than the L-shape (Wilcoxon signed Rank Test N = 13, Z = -3.042, p = 0.00; see Fig 6). Fig 6 indicates that the birds rarely needed more than two insertion attempts for all shapes except the Cross. Also, they rarely needed more than a single attempt to insert the Circle and interestingly the non-symmetric L-shaped key.

Importantly, subjects seemed to use shape-matching to solve keyset A (due to the keys’ geometrical properties, it was impossible to insert them any other way) but not Keyset B. Qualitatively, the birds initially seemed to attempt to align the axes of the object of keyset B but soon discovered alternative solutions to each different task which were then used uniformly to insert the correct key into its corresponding keyhole: the One-Arm key was held in a way that the protrusion either faced backwards or forwards while being inserted, the two protrusions correspondingly faced backwards and forwards in the Two-Arm condition; in the L-Shape condition subjects held the tool at one of the protrusions, inserted the other one into the box and tilted the object upwards through the hole; the Tripod was held at one end of its long axis and thus only the protrusion at its short axis was fitted through the one of the three remaining grooves; in the Cross condition, they proceeded similarly but aligned two opposite protrusions to their grooves (For more information, watch S1 and S2 Videos).

## Discussion

### Unrewarded shape matching

In the first part of our study, when birds were offered a selection of objects alongside various substrate cutouts in an unrewarded context, they combined objects with their corresponding cutouts at chance levels during the initial phase. Bringing a shape into contact with its corresponding frame thus seems to occur accidentally while trying to insert different objects into different substrates. The behaviour seems to be driven by these cockatoos’ playful propensity to combine two objects or objects with substrates in different ways. Previous studies indicate that such combinatory object play occurs at similar levels in this species as in habitually tool using birds such as New Caledonian crows and black Palm cockatoos [14,30]. Nevertheless, experience in unstacking objects and, even more, human demonstrations strongly increased the number of correct object-substrate combinations as well as successful shape-frame matches. This indicates that both experiencing the manipulative effect of unstacking an object from a pole/indention as well as the social enhancement involved in a human demonstration strongly increases the birds’ motivation to interact with the object on the corresponding pole/substrate. Thereby, the birds seem to attempt to playfully repeat the experienced/observed action despite the lack of a reward. In particular, the strong effect of demonstrations is surprising as a previous study showed little impact of stimulus enhancement by a conspecific on the choice of a non-food object for rostral manipulation in this species [31]. A human may be associated with food from previous experiments and thus enhance exploration. Alternatively, an object-substrate interaction may constitute a more interesting demonstration than a simple rostral manipulation. Incidentally, the birds have been shown to learn socially in a tool using context [29].

### Keybox

As soon as shape-frame matching became part of the solution to a tool using task in the keybox experiment, subjects became proficient at fitting the appropriate object into a corresponding opening, regardless of its shape.

#### Keybox: Tool selection

Our evaluation on tool selection signifies that subjects picked the correct key above chance expectation immediately, within the first session block, for both keysets. Nevertheless, they kept perfecting their performance throughout sessions in keyset B.

This is fairly surprising as the same subjects kept failing a basic matching to sample (MTS) task on the touchscreen for hundreds of trials in the previous year [20]. As they could solve different problems on the touchscreen it is unlikely that they had problems with vision or motivation [32]. Nevertheless, it is possible that the virtual environment of the touchscreen setup appeared too abstract to allow for fast MTS conceptualisation. As mentioned before, direct comparisons between touch screen tasks versus using solid objects indicate a similar trend in some birds and humans [21–23]. It is likely that having a haptic in addition to a visual experience and solid 3D versus 2D objects may increase the number of stimuli that can be used for discrimination [33, 34]. An even more practical explanation is that matching a tool into a corresponding substrate frame may provide a more purposeful context than merely learning to match same to same.

Interestingly, subjects performed better in condition 1 than condition 2 of keyset A. Here it is important to note that in condition 2, the remaining objects were not removed after a key was selected, allowing the birds to correct a wrong choice. The costs imposed by an incorrect selection in condition 2 were therefore much smaller than in condition 1. Furthermore, the parrots tend to lose interest after extensive exposure to the same experimental setups and start exploring the affordances of the apparatus instead of solving the problem in the habitual way, e.g. [30, 35, 36].

While making a selection, the animals often picked up one object, but discarded it in favour for another prior to insertion (note that we only removed the other objects in condition 1 after they had made contact with the apparatus). At least for keyset B, the second key the birds chose was usually the correct one. This makes it likely that the animals use a combination of haptic information from holding the key against their beak tip organ [37] as well as visual information from the keyhole in order to exclude specific objects for the task at hand: our subjects have previously shown to be able to use inference by exclusion in a more abstract setup [32]. Moreover, in a tool making study [17] they were observed to immediately discard material pieces that they had previously manufactured but that were of insufficient length to poke at an out-off-reach food after checking up on the distance of the goal item. The One-Arm keyhole produced particularly many of such object ‘switches’. One possible explanation for these results could be the lack of features provided by the One-Arm keyhole as opposed to the other keyholes featuring at least two grooves and therefore also more information about the correct key. In the case of the One-Arm keyhole, inference by exclusion might therefore have been a feasible strategy to find the correct key. Notably, in Demery’s study [26], asymmetrical shapes were explored more than symmetrical ones, suggesting that the asymmetrical One-Arm keyhole triggered more extensive exploration before insertion.

#### Keybox: Insertion effort

Evidently, frequency and duration are not independent (the more placement attempts a bird makes, the longer it takes to successfully insert the key). Therefore, finding trends in the same direction is not unexpected.

Data on frequency in keyset A suggests that subjects constantly inserted the circular object on the first placement attempt and that they needed only around 2 placement attempts for both the Square and the Triangle. This is in contrast to previous findings in primates, which needed around 4–5 placements on average when inserting a stick into a circular hole or a stick into a groove of the same length (the capuchins needed 9 placements for the latter) [7]. However, our results parallel findings in human infants, who were more successful in inserting objects with a lower level of symmetry [1].

Nevertheless, data on frequency and duration in keyset A parallels primate results and, unsurprisingly, confirms our predictions that lower levels of symmetry seem to increase the animals’ insertion effort.

As to the question whether some keys might have been easier to grasp than others due to their shape (keys with more edges might be easier to hold, e.g. the Square easier than Triangle), frequency and duration do not show a significant difference. Due to the highly coordinated beak-tongue movements parrots are capable of, the key shape is rather unlikely to have an influence on the birds’ grasp.

Findings on keyset B, however, contradict predictions that would be made according to the different degrees of symmetry and number of axis on the objects: If the objects had been fully aligned as in keyset A in order to achieve insertion we would have expected the birds’ insertion effort to behave as follows: L-Shape>Tripod>One-Arm>Two-Arm> Cross. Instead, what we found was this trend: Cross>Tripod>Two Arm>L-shape & One Arm. These patterns can be explained by the animals’ object-specific insertion techniques to circumvent alignment of perforating object features with the keyhole. This was possible as we decided not to use objects that had elongated depth (as the upper mandible is bigger and curved, we were not sure whether the birds would still be able to have full sight of the keyhole during insertion with the tool pointing down their beak). Furthermore, if the surface of the depth became larger than the surface of the front subjects might pay less attention to the shape of the front. The parrots turned each object in a specific manner, consequently avoiding the alignment of some of the features by holding at least one protrusion and, if possible, two, facing forwards and backwards during insertion (see S2 Fig, S1 and S2 Videos). Hence, they only needed to align one remaining protrusion when the Tripod was the key and two remaining protrusions when the Cross was the key, explaining their above performance.

Additionally, subjects behaved flexibly, using a completely different motor routine for inserting the L-shape, inserting first one of the two protrusions into the big part of the opening and thereafter tossing the rest of the object through the keyhole with an upward movement of the beak. This made the insertion of the L-shape key as successful as the insertion of the One-Arm. As mentioned previously, human infants facing a posting task featuring a disc and a linear slot for insertion (as inserting a coin into a cash machine), did not turn the disc to facilitate insertion before 24 months of age [2]. This finding is partly linked to the poor development of an allocentric frame of reference in younger infants. The birds, in contrast, seem to quickly learn to pay attention to the major axes of the objects as well as the insertion groove. Also, the parrots turning the objects to avoid insertion effort suggests that they do seem to use an allocentric frame of reference when handling these tools.

We finally looked at a possible effect of playful experience with unrewarded inserting and stacking/unstacking of similar object shapes on performance in both tool selection as well as insertion effort. Although all birds in the test group had successfully inserted/stacked/unstacked each shape several times before testing started, we failed to find any effect of unrewarded shape matching experience on birds’ performance in the keybox task. Thus, the intense and intrinsically structured combinatorial object play in this species may not have a direct benefit, like gaining experience for future problem-solving tasks, but could simply be a side effect of their explorative foraging technique.

Otherwise, the objects used in the play experience may not have been sufficiently analogous in size and weight to the objects used in the test or that the experience was not intense enough to create an observable effect. Furthermore, the sequence of the tasks as well as the time passed between play experience and tool use task could have influenced the effect of play on the keybox task.

## Conclusion

Our results suggest that the ability to align objects to a corresponding substrate groove is neither limited to primates nor to habitually tool using species nor to animals with hand-like appendices. While our animals seem to use an allocentric frame of reference to achieve object insertions it is not yet possible to determine whether they construct the alignments in 3 dimensions as we were unable to observe small details of the adjustment of the object on the substrate with the birds blocking the view too often to obtain reliable data.

Shape-frame matching abilities for capuchins and chimpanzees are likely to facilitate tool use. For example, in both species various populations have been observed to use sticks to probe for food in different holes and crevices, and to crack nuts by placing the nut on an anvil and then striking the nut with a hammer stone, e.g. [38–40]. The Goffin's cockatoo cannot be considered a habitual tool user, at least not on a population-wide level as suggested by unpublished data from Osuna-Mascaro & Auersperg. Nevertheless, they are highly opportunistic foragers using different feeding techniques that include extractive foraging according to unpublished data from O’Hara & Mioduszewska. While lacking primate hands, their beak is highly dexterous, with a flexible upper mandible and a tongue that can be moved in an almost thumb-like manner [30]. Additionally, Auersperg et al. [13] and unpublished data from Osuna-Mascaro & Auersperg suggest that they have a strong predisposition to combine objects during play and occasionally also during foraging. From present knowledge, we can assume that their ability to align objects seems to derive from domain general flexibility rather than a specific specialisation.

Future comparative research on the basics of object alignment should incorporate more avian species; particularly habitual and non-habitual tool using birds and nest building parrots would be of great interest. Follow up research on the Goffin's cockatoo will focus on fine details of object alignment and beak-tongue actions on the object before and during insertion which we hope to achieve by filming from the inside of a Perspex box. This will help us determine a better general overview of their fitting techniques and to evaluate the role of visual adjustment in these techniques [e.g. 9].

## Supporting information

S1 Video Insertion techniques in keyset A. (MP4)

S2 Video Insertion techniques in keyset B. (MP4)

S1 Fig Examples of shape/frame matching. Observed during Auersperg et al. 2014 (see reference list). Left: shape-frame matching; right: frame-shape matching.
(TIF)

S2 Fig Keybox. A) Inspection phase; B) Choice phase; C) Insertion; D) Platform collapses, food is released.
(TIF)

S3 Fig Play: Mean number of combinations of objects with substrate grooves. Combinations that were fitting the object in question (correct), within corrects, combination in which the object was successfully fitted (shape match) and combinations of objects with different shapes (wrong). Data is shown for each phase (1–3) within each playset (A-C).
(TIF)

S4 Fig Selection: Mean number of correct choices per trial for keysets A and B showing each key. keyset A (1 = Square, 2 = Circle, 3 = Triangle), keyset B (1 = One-Arm, 2 = Two-Arm, 3 = L-Shape, 4 = Tripod, 5 = Cross). Chance expectation for keyset A = 0.33; for keyset B = 0.2 correct per trial. * chosen above chance expectation; ** p<0.0001.
(TIF)

S1 Table Subjects’ sex, year of hatching and the group division. (TIF)

S2 Table Inter-observer reliability results. Intra-Class Correlation Coefficient (ICC).
(TIF)

S3 Table Results GLMM analysis for USM. Listed for each playset (A-C) for each target (no. of correct combinations; number of shape matches). Fixed factors are condition (1–3); object and session; random factor is subject. Above chance p-values are marked in yellow.
(TIF)

S4 Table Individual data (number of correct choices) for each subject of each group. (T = enhanced play experience; C = no enhanced play experience) for each keyset (A & B) for each condition (1&2). For keyset B, the data are further divided into two session blocks for each condition: * significantly above chance (Binominal test); ** p<0.0001.
(TIF)

S5 Table Results GLMM analysis for keybox selection. Listed for each keyset A and B on no. of correct objects selected. Fixed factors are condition (1–2); key (1–3 in keyset A, 1–5 in keyset B) session; group and an interaction group*key; random factor is subject. Above chance p-values are marked in yellow.
(TIF)

S6 Table Results GLMM analysis for keybox switches. Listed for each keyset A and B on no. of correct objects selected. Fixed factors are condition (1–2); key (1–3 in keyset A, 1–5 in keyset B) session; group and an interaction group*key; random factor is subject. Above chance p-values are marked in yellow.
(TIF)

S7 Table Individual data on the total number of object switches before combining an object with apparatus. Also listed is the number of times the object switched for was the correct choice. * significantly above chance (Binominal test); ** p<0.0001.
(TIF)

S8 Table Results GLMM analysis for the keybox insertion effort. Listed for each keyset A and B on duration and frequency as target variables. Fixed factors are condition (1–2); key (1–3 in keyset A, 1–5 in keyset B) session; group and an interaction group*key; random factor is subject. Above chance p-values are marked in yellow.
(TIF)

S1 Dataset Complete dataset. (XLSX)
