# Supplementary figures and images for: The keybox: Shape-frame fitting during tool use in Goffin’s cockatoos (Cacatua goffiniana)
Source: PLoS One. 2017 Nov 8;12(11):e0186859. doi: 10.1371/journal.pone.0186859 (PMC5695585; doi:10.1371/journal.pone.0186859)

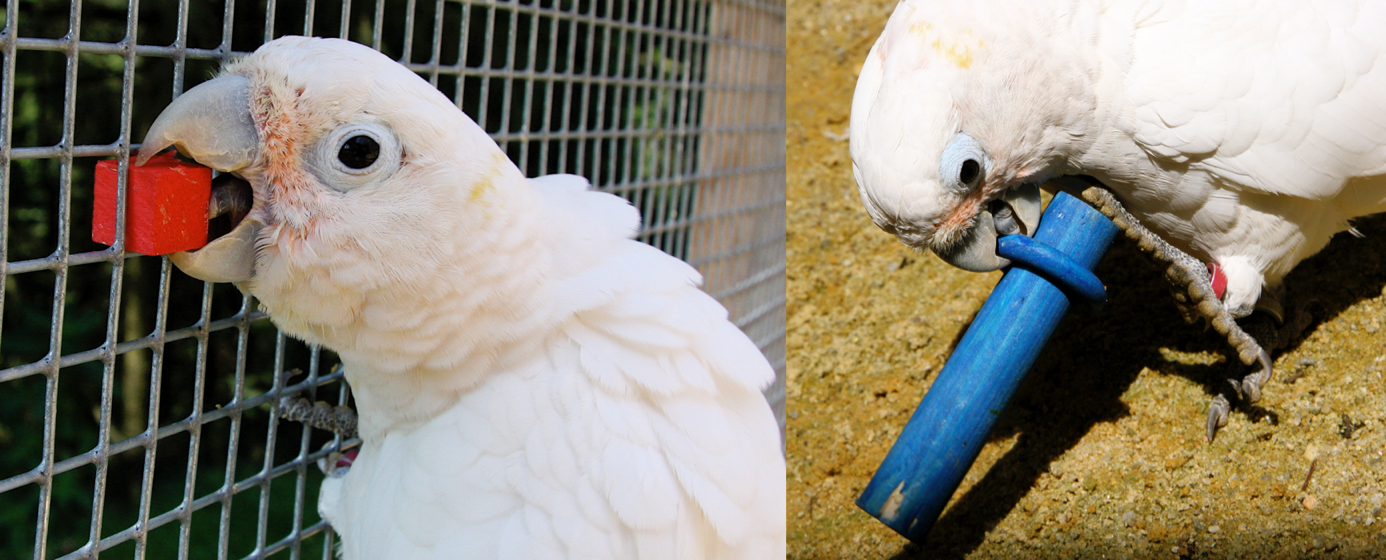

Supplement: S1 Fig — Observed during Auersperg et al. 2014 (see reference list). Left: shape-frame matching; right: frame-shape matching. (TIF) [file pone.0186859.s003.tif]

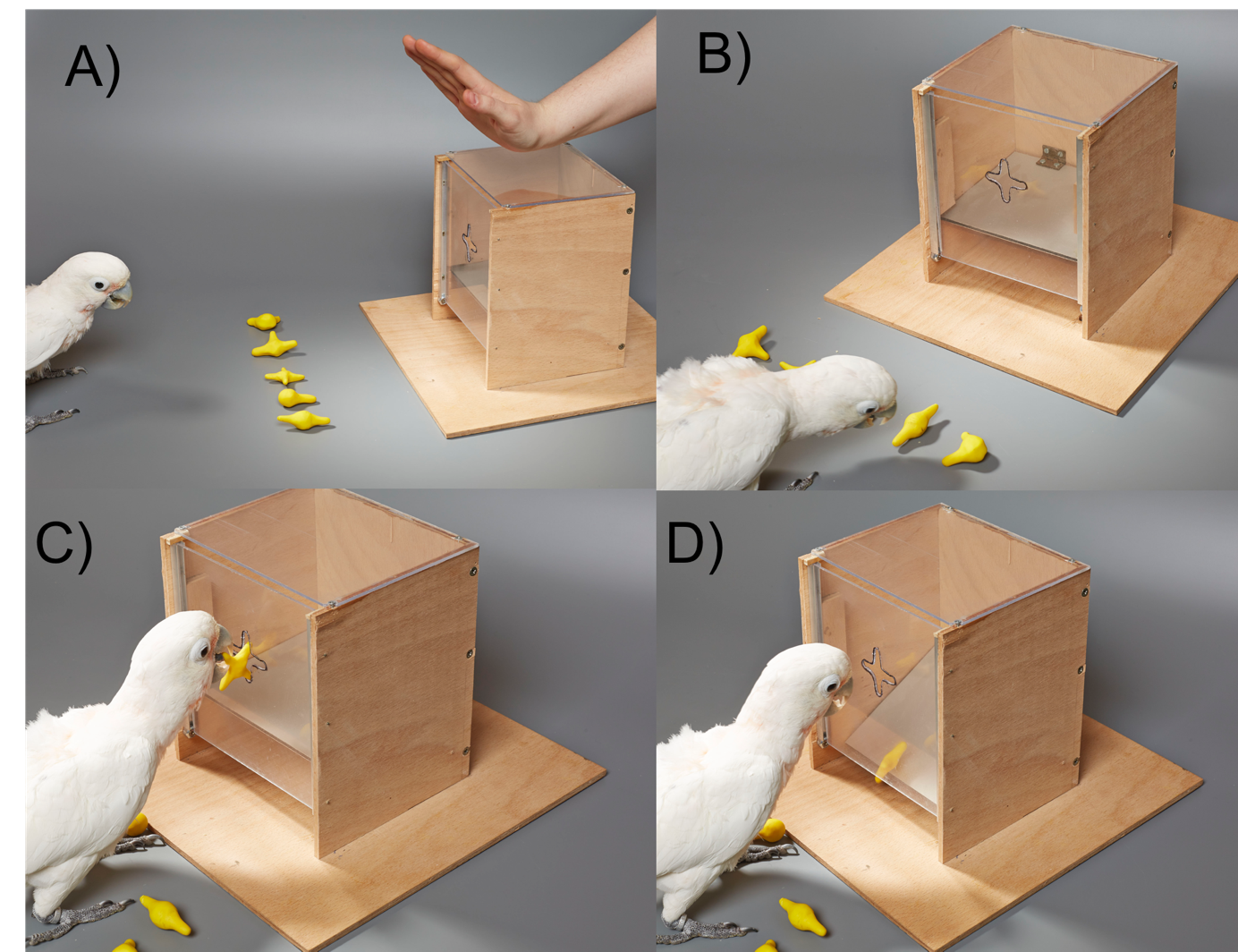

Supplement: S2 Fig — A) Inspection phase; B) Choice phase; C) Insertion; D) Platform collapses, food is released. (TIF) [file pone.0186859.s004.tif]

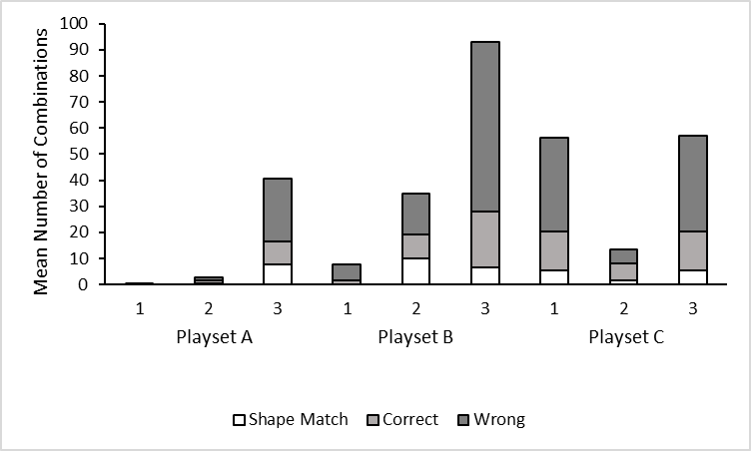

Supplement: S3 Fig — Combinations that were fitting the object in question (correct), within corrects, combination in which the object was successfully fitted (shape match) and combinations of objects with different shapes (wrong). Data is shown for each phase (1–3) within each playset (A-C). (TIF) [file pone.0186859.s005.tif]

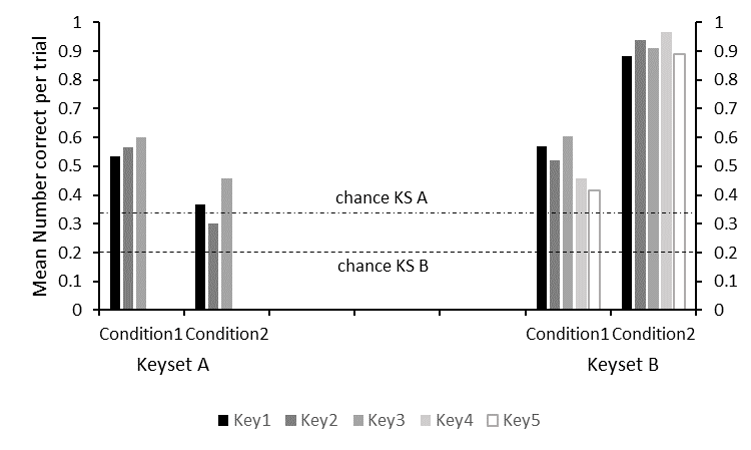

Supplement: S4 Fig — keyset A (1 = Square, 2 = Circle, 3 = Triangle), keyset B (1 = One-Arm, 2 = Two-Arm, 3 = L-Shape, 4 = Tripod, 5 = Cross). Chance expectation for keyset A = 0.33; for keyset B = 0.2 correct per trial. * chosen above chance expectation; ** p<0.0001. (TIF) [file pone.0186859.s006.tif]

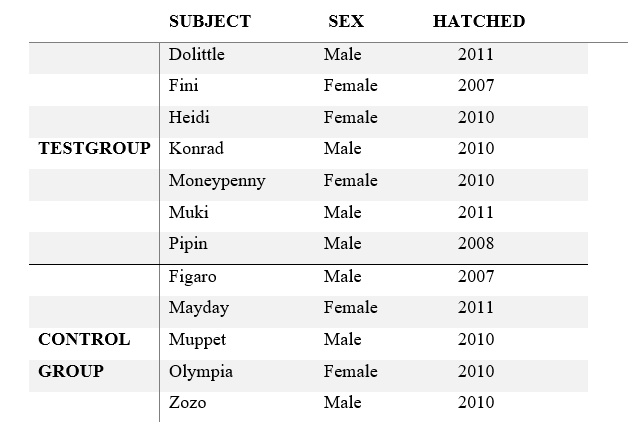

Supplement: S1 Table — (TIF) [file pone.0186859.s007.tif]

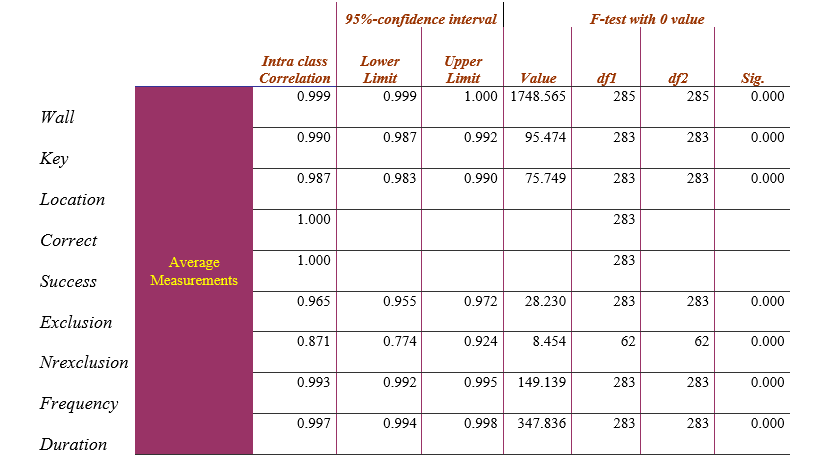

Supplement: S2 Table — Intra-Class Correlation Coefficient (ICC). (TIF) [file pone.0186859.s008.tif]

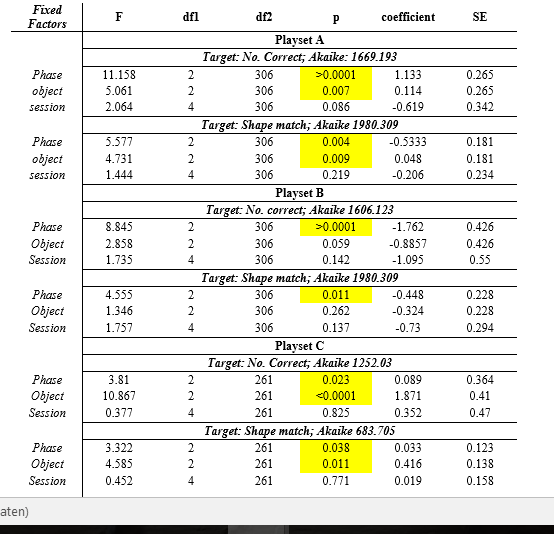

Supplement: S3 Table — Listed for each playset (A-C) for each target (no. of correct combinations; number of shape matches). Fixed factors are condition (1–3); object and session; random factor is subject. Above chance p-values are marked in yellow. (TIF) [file pone.0186859.s009.tif]

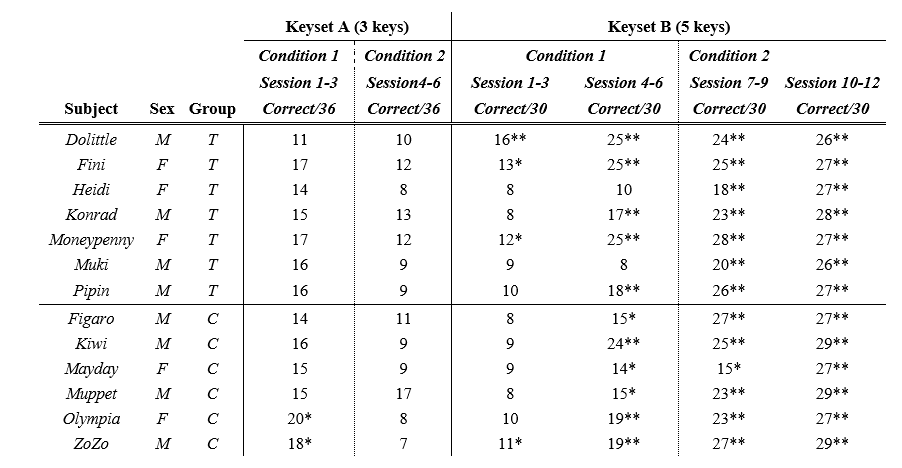

Supplement: S4 Table — (T = enhanced play experience; C = no enhanced play experience) for each keyset (A & B) for each condition (1&2). For keyset B, the data are further divided into two session blocks for each condition: * significantly above chance (Binominal test); ** p<0.0001. (TIF) [file pone.0186859.s010.tif]

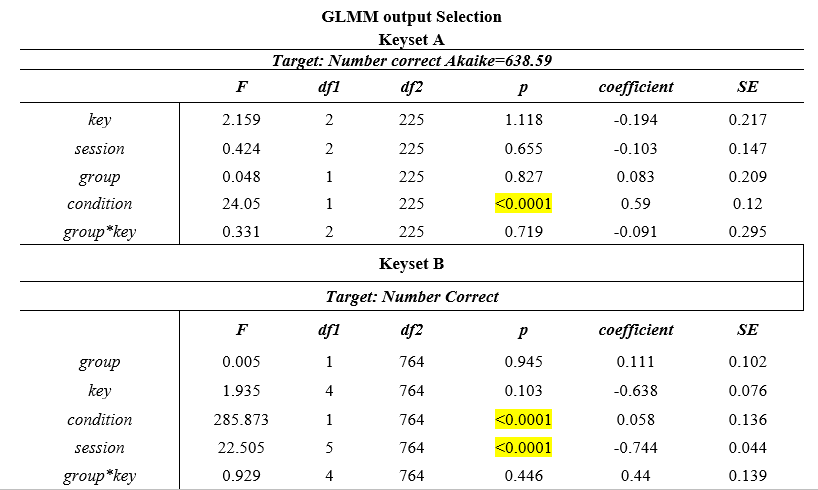

Supplement: S5 Table — Listed for each keyset A and B on no. of correct objects selected. Fixed factors are condition (1–2); key (1–3 in keyset A, 1–5 in keyset B) session; group and an interaction group*key; random factor is subject. Above chance p-values are marked in yellow. (TIF) [file pone.0186859.s011.tif]

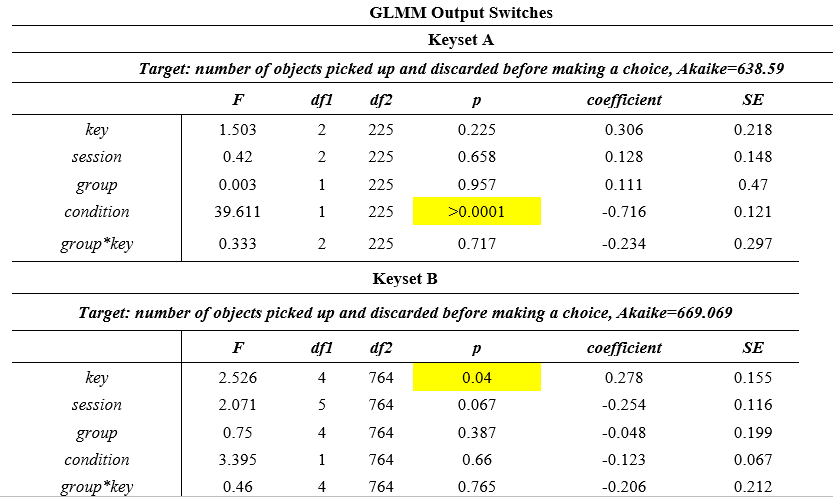

Supplement: S6 Table — Listed for each keyset A and B on no. of correct objects selected. Fixed factors are condition (1–2); key (1–3 in keyset A, 1–5 in keyset B) session; group and an interaction group*key; random factor is subject. Above chance p-values are marked in yellow. (TIF) [file pone.0186859.s012.tif]

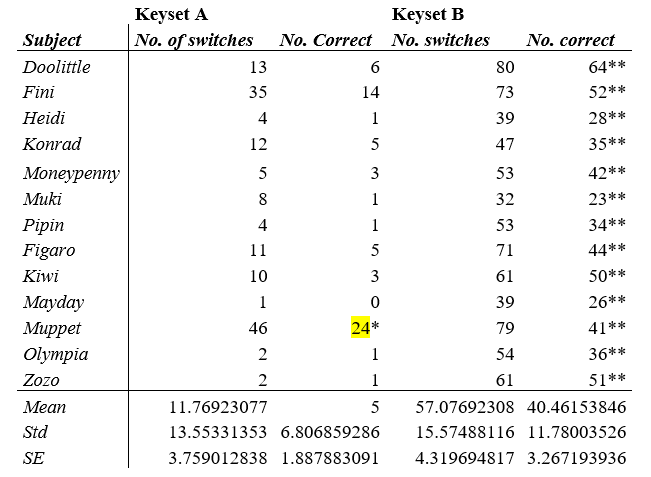

Supplement: S7 Table — Also listed is the number of times the object switched for was the correct choice. * significantly above chance (Binominal test); ** p<0.0001. (TIF) [file pone.0186859.s013.tif]

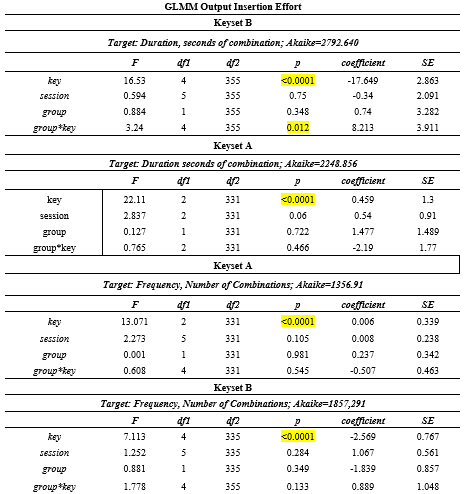

Supplement: S8 Table — Listed for each keyset A and B on duration and frequency as target variables. Fixed factors are condition (1–2); key (1–3 in keyset A, 1–5 in keyset B) session; group and an interaction group*key; random factor is subject. Above chance p-values are marked in yellow. (TIF) [file pone.0186859.s014.tif]
